# Large-Scale Water Quality Prediction Using Federated Sensing and Learning: A Case Study with Real-World Sensing Big-Data

**DOI:** 10.3390/s21041462

**Published:** 2021-02-20

**Authors:** Soohyun Park, Soyi Jung, Haemin Lee, Joongheon Kim, Jae-Hyun Kim

**Affiliations:** 1 School of Electrical Engineering, Korea University, Seoul 02841, Korea; soohyun828@korea.ac.kr (S.P.); jungsoyi@korea.ac.kr (S.J.); haemin2@korea.ac.kr (H.L.); 2 Department of Electrical and Computer Engineering, Ajou University, Suwon 16499, Korea

**Keywords:** federated learning, smart IoT sensor, big data, optimization, scheduling

## Abstract

Green tide, which is a serious water pollution problem, is caused by the complex relationships of various factors, such as flow rate, several water quality indicators, and weather. Because the existing methods are not suitable for identifying these relationships and making accurate predictions, a new system and algorithm is required to predict the green tide phenomenon and also minimize the related damage before the green tide occurs. For this purpose, we consider a new network model using smart sensor-based federated learning which is able to use distributed observation data with geologically separated local models. Moreover, we design an optimal scheduler which is beneficial to use real-time big data arrivals to make the overall network system efficient. The proposed scheduling algorithm is effective in terms of (1) data usage and (2) the performance of green tide occurrence prediction models. The advantages of the proposed algorithm is verified via data-intensive experiments with real water quality big-data.

## 1. Introduction

Environmental pollution has been one of the most serious problems for a long time and the situation is getting worse due to the development of modern industrial societies. Human beings have suffered from a series of problems, such as diseases, ecosystem destruction, and natural disasters caused by the polluted environment [1,2,3,4,5,6,7,8]. Especially, for water pollution, contaminated water directly affects human life from food poisoning to the deaths caused by microbes and metallic substances that exist in water. Green tide phenomenon, which has been mentioned as one of more serious water pollution problems in this paper, is a typical water pollution that has frequently occurred worldwide [9,10,11]. The green tide causes problems which are directly related to human health and the destruction of underwater ecosystems due to the overpopulation of algae. In addition, according to the report by the World Health Organization (WHO), it has been reported that more than 3.4 million people die due to polluted water-related diseases every year [9]. Furthermore, the degree of water pollution becomes more serious by continuous development and industrialization.

Efforts to detect changes in water quality and predict the patterns of water quality have changed to prevent pollution, and they are modeled and estimated in various forms using Internet of Things (IoT)-based smart sensors [12,13,14,15,16,17]. In the early traditional water quality monitoring study results, laboratory-based research using sample data was common. However, this approach obviously has limitations on the reflection of real-time real-world data sampling when the data become complicated and larger. In order to overcome the limitations, machine learning (ML) and deep learning (DL)-based research has also been conducted, which is one of possible solutions to effectively analyze various environmental indicators by gathering data from IoT sensors and also to derive the statistical relationship between the received data from sensors and water quality [18,19,20].

As discussed in [19], there are several previous related research results that apply ML/DL algorithms to water quality modeling and estimation. However, the models are reasonable and sufficiently accurate at specific times and places. Therefore, it is obviously required to design new models based on up-to-date sampling data, because the existing models are only valid in previous time sampling data.

For this reason, this paper proposes a new water quality monitoring system which is designed for achieving federated learning (FL) functionalities. The system is fundamentally based on distributed network architectures, and it consists of smart IoT sensors, edge servers, and a central cloud. The edge server gathers IoT sensing data, learns a ML/DL-based prediction model using real-time real-world sensing data and communicates with the centralized cloud, and the cloud builds and updates a global prediction model for green tide occurrence, which can be applied to a wide range of regions.

**Figure 1:**
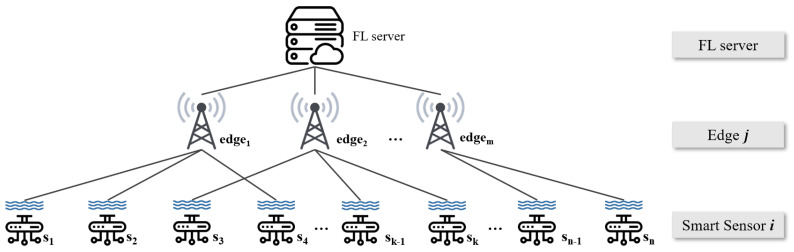
Overall network architecture.

Instead of FL computation, which is for conducting ML/DL learning via distributed computing platforms and then aggregating the results in a centralized platform to build a global model, centralized learning is optimal in terms of learning accuracy [2,21]. However, having all data in a centralized platform is not easy. Especially with this water quality monitoring problem, it is not realistic to assume that all sensing data can be gathered in a centralized platform simultaneously, due to geologically physical distances. This issue can be solved by utilizing a number of edge servers (i.e., local distributed computing platforms in FL) that are distributed in a slightly closer position to the sensors [21]. In addition, a global model which aggregates several model parameters from multiple distributed edge servers can be efficiently and effectively built via FL without sharing all sensor data with the central server. In fact, FL computation allows for the global model to derive performance close to central computing while reducing the communication cost of the entire networking systems [22,23,24]. For this reason, we design a network system which consists of multiple edges, distributed smart sensors, and a central server. Therefore, our network architecture can be illustrated as shown in Figure 1.

In this system, a number of smart sensors are distributed in large areas to collect various water quality-related indicators in the rivers and lakes of South Korea. The edges that are scattered around the sensors in order to receive data from the smart sensors are matched by the scheduling algorithm between edges and smart sensors. The transmitted data from various locations are used to learn/train the green tide prediction model. According to the progress of FL computation, the performance of local models that participate in aggregation affects the performance of the global model generated in the central cloud. We decided that only edge servers that guarantee the model’s accuracy can participate in the aggregation, and their parameter information is utilized. In this process, maximizing the number of edge servers that have models that guarantee the accuracy of the green tide occurrence forecast is important. Therefore, we design a fairly optimal scheduling algorithm for this purpose in order to maximize the number of local edge learning initiations. When multiple edge servers receive data from distributed smart sensors, the proposed scheduler lets as many edges as possible have sufficient data for learning without significant gaps in the amount of data supplied between each edge. It is meaningful in an environment where there is a difference in the degree of dispersion of smart sensors around the actual position of edges or where certain edges can monopolize the matched sensor data. In addition, setting a baseline (i.e., training initiation threshold) for appropriate learning data can prevent some cases that affect the performance of the green tide occurrence prediction models, such as overfitting and underfitting caused by the insufficient amounts of data.

The main contributions of this paper are as follows:

- The existing research results that use only limited case information at specific times and places are not suitable for the accurate prediction of green tide phenomenon. The general central computing method is not suitable to handle massive real-time water quality data from the several smart sensors that are located over the river irregularly. A new network model with a distributed structure, which is based on FL is suggested. Based on the network model, we can utilize real-time data generated at a certain frequency for a general prediction global model rather than for a certain point.
- This paper presents an optimal fair scheduling algorithm for efficient data transmission between edge servers and smart sensors. The algorithm prevents some edges to receive excessive data from the smart sensors and allows as many edge servers to perform local model training by maximizing the number of learning edges where the learning edge is defined as the edge that has sufficient data to initiate prediction model leaning/training (i.e., more than the threshold *D*).
- For data-intensive simulations, actual real-world domestic water quality big-data is used. The river in the area where green tide occurs in Korea is designated as the background of the experiment. Water quality data managed and provided by several Korean government-affiliated organizations is used for the performance evaluation in this paper.

The rest of this paper is organized as follows. Section 2 illustrates related work and considers our reference network model. Section 3 presents our proposed FL scheduler for managing sensing data in large-scale network environment. Section 4 evaluates the performance of our proposed algorithm with real-world dataset. Lastly, Section 5 concludes this paper and presents future research directions.

## 2. Preliminaries

In this section, prior research results related to the proposed algorithm and the corresponding reference network architecture are described.

### 2.1. Related Work

Remarkable research results for water quality management and monitoring have been proposed, especially for green tide phenomenon [12,13,14,15,16,17,18]. The precise and accurate case study results for various sensor network and vehicular network situations are presented in [12,13,14,15]. The smart sensor design for water quality monitoring is precisely discussed in [16,17], In [18], a drinking-water quality model is proposed to predict water quality using big data with DL methodologies by utilizing long short-term memory (LSTM) for time-series prediction. The drinking-water quality data measured by the automatic water quality monitoring stations of Guazhou Water Source of the Yangtze River in Yangzhou are used to analyze the water quality parameters in detail, and finally, the prediction model is trained and tested with monitored real-world data from January 2016 to June 2018. The results of the study present that the prediction values of several water quality indicators and the actual values are almost equivalent. The proposed algorithm in [18] predicts the trends of measurement variations in several water quality indicators. The drinking-water is managed by comparing the prediction values and reference values of each component determined by the water quality. However, this model is built after gathering all data, thus it is not for real-time computation. Instead of this, our proposed method in this paper uses distributed learning in order to gather the data in real time, and also trains them in local for build local models in real-time and, finally, the local models are used to compute a global model via FL functionalities where the learning is conducted via our proposed fairly optimized scheduler to improve the performance.

### 2.2. Reference Network Model

**Figure 2:**
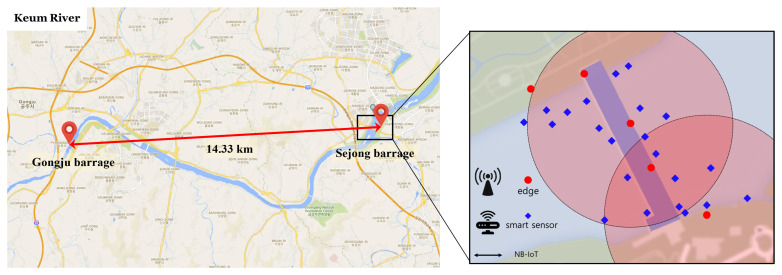
Overall network architecture: This shows Gongju barrage and Sejong barrage in the Keum River, where the actual green tide is found in Korea are considered in this paper. Several edges and smart sensors are distributed around one barrage and are designed to communicate through NB-IoT. The red dot shown in the figure is edge located on the barrage and the blue diamond is smart sensor that measures water quality in the river.

In this research, many smart IoT-based networked sensors are distributed in riversides. In order to conduct the research, which reflects real-world data and scenarios, one of the major rivers in South Korea is considered, i.e., Keum river, as illustrated in Figure 2. According to the fact that the river is nation-wide, one-hop sensing data transmission from sensors to the centralized DL computing platform is not possible because (1) transmission distances will be in the order of kilometers, as shown in Figure 2 and (2) having massive medium access at a single access point (i.e., centralized computing platform) with numerous smart sensors that are deployed in the entire river is not efficient at all in terms of network throughput degradation. Therefore, edge servers are essentially required in order to gather the water quality sensing data at first. The edge servers are able to locally conduct DL training computation; then, the trained local DL model parameters can be delivered to the centralized computing platform. The reason why edge servers are used not only for sensing data gathering but also for local training is that conducting local training in individual edge servers is beneficial in terms of learning computation robustness. If the centralized computing platform gathers all data themselves from all edges servers, the amounts of data will be huge and the sensing data arrivals at the centralized computing platforms will not be synchronized. Thus, the centralized computing platform will have arrivals while the deep learning training computation is still working one. This situation is harmful in terms of robust training. In considering FL computation in this paper, local training in individual edge servers will compute optimized local training parameters in each edge server. After that, the trained parameters in individual edge servers will be delivered to the centralized computing platform in each synchronized unit time, then the simple aggregation computation will be performed in a short time at the centralized platform. Finally, we can observe that this local training-based FL computation is beneficial in terms of robust training. The centralized platform can perform FL computation in order to build our desired global DL model that can predict green tide phenomenon. Therefore, it is obvious that FL functionality is essentially required for this nationwide large-scale sensing and monitoring system.

In the river, multiple barrages exist, as illustrated in Figure 2. The barrages are located in suitable places to set up multiple edge servers and smart IoT-based water quality monitoring sensors. The reason is that the barrages can provide power sources to edge servers and the sensors should be located nearby the edge servers for more reliable transmission of sensed water quality data. The example geometric location figure is presented in Figure 2, where the edge servers and sensors are denoted by red circles and blue diamonds, respectively. Note that the water quality data gathered by smart IoT-based sensors are used to determine whether the green tide phenomenon does or does not occur in the river.

For the transmission from smart IoT-based sensors to edge servers, narrow-band IoT (NB-IoT) communications are used for establishing massive connections between sensors and edge servers in large-scale networks [25]. As discussed in [25], NB-IoT allows for the connectivity of more than 100 K devices per cell and it could be increased by exploiting multiple carriers, and the maximum throughput rate is 200 and 20 kbps in downlink and uplink, respectively, with a 1600-byte payload size in each message. Moreover, the communication range of NB-IoT is about 10 Km [26]. Furthermore, the use of NB-IoT is also beneficial in terms of energy efficiency, thus it is definitely helpful for battery-limited smart IoT-based sensors (e.g., 10 years of battery lifetime).

The water quality observation data generated by smart sensors are used as learning data to create a green tide prediction model in each local edge server. The smart sensor means a new IoT-based networked sensor that incorporates various sensors for green tide indication, i.e., temperature, pH, dissolved oxygen (DO), total organic carbon (TOC), total nitrogen (TN), Chlorophyll-a (Chl-a), and turbidity.

## 3. Scheduler Design for Federated Learning

In this section, we describe the motivation to design the optimal fair scheduling and the details of the proposed algorithm.

### 3.1. Algorithm Design Rationale

**Figure 3:**
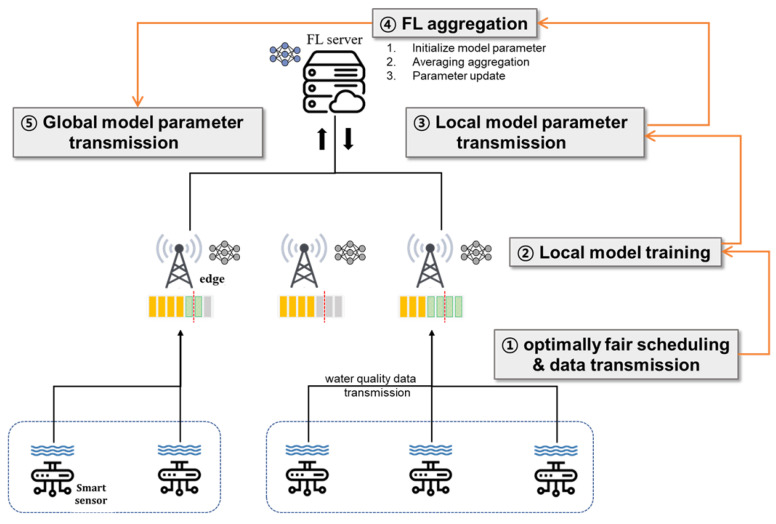
Flowchart of the overall system.

According to the scheduling policies for the communication matching between edge servers and smart sensors, the numbers of associated sensors in edge servers can be varied. In general, after matching is complete, the edge servers perform their own local training for green tide prediction based on real-world sensing data. If some edge servers are with an insufficient number of water quality sensing data, the performance will be poor due to overfitting. Eventually, this result is definitely harmful for the learning accuracy performance of our ultimate global prediction model because the model will be built by the aggregation of local prediction models through the FL model aggregation procedure as illustrated in Figure 3 [27]. First of all, local edge servers gather sending data in order to conduct local model training based on that. For the gathering, scheduling decision for matching between edge servers and sensors should be made in terms of optimal fair scheduling in order to build accurate global model eventually (Step 1). After gathering all the data, each edge server conducts local model training (Step 2) and Algorithm 1. Then, the local model training results (i.e., learning parameters) should be delivered to centralized platform for FL computation via local model parameter aggregation in order to build our final global model (Step 3 and Step 4) and Algorithm 2. The global model, which is generated by FL computation, is distributed to edge servers (Step 5).

Based on this nature of FL computation, it is important to ensure that the edges have sufficient numbers of training data to satisfy a certain level of local model performance. Therefore, our proposed scheduling algorithm is designed for maximizing the number of edge servers those training data are sufficient to conduct training. Here, the required number of training data for our target performance is defined as a baseline (i.e., training initiation threshold). It is obvious that the training initiation threshold can be varied depending on the required target performance of our green tide prediction model. Our proposed optimally fair scheduling algorithm for FL computation in distributed network is able to avoid training data imbalance among edge servers. Furthermore, the algorithm is able to control certain edges to proceed with local learning with a quantity of data that cannot satisfy the target performance. Through this solution, the performance degradation by overfitting or underfitting in some edge servers can be avoided.
**Algorithm 1** Fair Federated Learning Computation at Edge Server *i*.1:**Input:** Sensing Data: $s_{i}$2:**Output:** Local Learning Parameters: $w_{i}$3:Local_Training(Edge ID: *i*, Sensing Data: $s_{i}$):4:**for** For given training iteration **do**5: $w_{i}\leftarrow w_{i}-\alpha ▽L\left(w_{i},s_{i}\right)$ // L(·): Cost Function, α: Learning Rate6:**end for**
**Algorithm 2** Fair Federated Learning Computation at a Central Platform.1:**Input:** Local Learning Parameters: $w_{i}$, ∀i∈E // E: Set of Edges2:**Output:** Global Learning Parameter: $w_{G}$3:$w_{G}=\frac{1}{\left|E\right|}\sum_{\forall i\in E}w_{i}=\frac{1}{\left|E\right|}\sum_{\forall i\in E}\mathrm{Local}\_\mathrm{Training}(i,s_{i})$

### 3.2. Edge Queue Model

In order to formulate our proposed method, it is required to model the queue in each edge server. Each edge stores water quality data in the internal queue and use the stored data to start local model training. The queue $Q_{j}\left[t\right]$ is formulated as follows,

(1) $$Q_{j}\left[t+1\right]=\left(1-I_{j}\right)\cdot \left(Q_{j}\left[t\right]+\sum_{\forall s_{i}\in S}d_{i}^{s}\left[t\right]\cdot x_{\left(i,j\right)}\left[t\right]\right),\forall e_{j}\in E$$

where $Q_{j}\left[t\right]$ in (1) stands for the saved water quality data size of edge $e_{j}$ at *t* where the set of edges is denoted as E. In addition, $s_{i}$, S, $d_{i}^{s}\left[t\right]$, and $x_{\left(i,j\right)}\left[t\right]$, stand for smart sensor *i*, set of sensors, the amount of transmitted data from sensor *i* at *t*, and scheduling vector between edge *j* and sensor *i* at *t* where it can be 1 it they are scheduled (otherwise, 0), respectively. The size of the queue is independent to the training initiation threshold *D*, and has an infinite size. These features allow the edge to store all data in a queue even if it receives data larger than the specified *D* size in one iteration and to use the stored data for learning on the edge. The queue is controlled by a scheduling vector $I_{j}$ that will be explained again, the queue is emptied only when edge starts local learning using the data that has met the training initiation threshold *D* (i.e., $I_{j}$ = 1). If the size of the data received at iteration *t* does not meet the condition (i.e., $I_{j}$ = 0), according to (1), the edge accumulates the data transmitted at iteration *t* until the data stored in the queue is able to meet the size of *D*.

### 3.3. Algorithm Details

**Figure 4:**
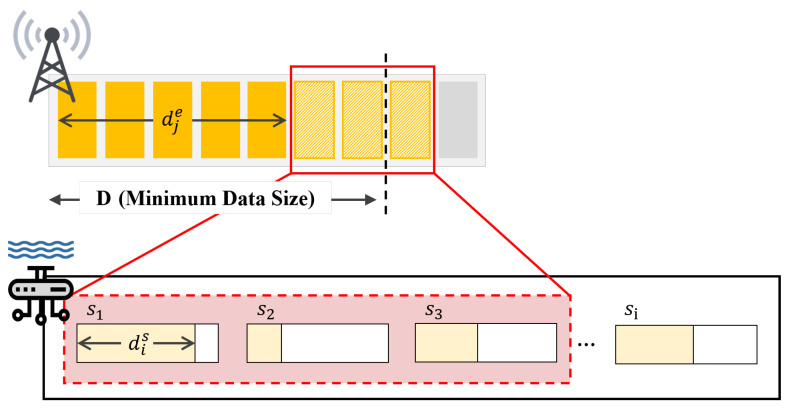
Optimally fair FL scheduling algorithm.

The proposed fair FL scheduling algorithm for green tide prediction with water quality sensing training data is presented as follows. Note that the proposed algorithm individually operates in each river, and the overall network architecture is as illustrated in Figure 4.

(2) $$\begin{array}{ccc} \max: & & \sum_{\forall e_{j}\in E}I_{j}\left[t\right], \end{array}$$

(3) $$\begin{array}{ccc} \mathrm{subject}\;\mathrm{to} & & \sum_{\forall s_{i}\in S}d_{i}^{s}\left[t\right]\cdot x_{\left(i,j\right)}\left[t\right]\geq \left(D_{j}-d_{j}^{e}\left[t\right]\right)\cdot I_{j}\left[t\right],\forall e_{j}\in E, \end{array}$$

(4) $$\begin{array}{ccc} & & \sum_{\forall e_{j}\in E}x_{\left(i,j\right)}\left[t\right]\leq 1,\forall s_{i}\in S, \end{array}$$

(5) $$\begin{array}{ccc} & & \sum_{\forall s_{i}\in S}x_{\left(i,j\right)}\left[t\right]\leq a_{j},\forall e_{j}\in E, \end{array}$$

where $e_{j}$ and $s_{i}$ stand for edge servers *j* and smart sensors *i*, $\forall e_{j}\in E$ (set of edge servers) and $\forall s_{j}\in S$ (set of smart sensors), as illustrated in Figure 1, respectively. Then, $I_{j}\left[t\right]$ is a scheduling vector for the local model training initiation in $\forall e_{j}\in E$ at time *t*, i.e., $e_{j}$ is ready to go for local model training when $I_{j}\left[t\right]=1$ (otherwise, $I_{j}\left[t\right]=0$). Note that each $e_{j}\in E$ initiates its own local model training when it has enough training data, i.e., the number of sensing data is equal to or larger than $D_{j}$ (i.e., training initiation threshold). In addition, $x_{\left(i,j\right)}\left[t\right]$ is a scheduling vector between $e_{j}\in E$ and $s_{i}\in S$ at time *t*. Thus, we have to obtain optimal $I_{j}\left[t\right]$ and $x_{\left(i,j\right)}\left[t\right]$ via the optimization solving procedure where $e_{j}\in E$ and $s_{i}\in S$. Here, $d_{i}^{u}\left[t\right]$ and $d_{j}^{e}\left[t\right]$ stand for the existing sensing data amounts in $s_{i}\in S$ and $e_{j}\in E$ at time *t*, respectively. In (3), $D_{j}$ stands for the data size, which is required for initiating local model training in $e_{j}\in E$. Lastly, $a_{j}$ stands for the number of antennas in $\forall e_{j}\in E$.

As mathematically described in this optimization formulation (2)–(5), our main objective is for maximizing the number of edges, which can initiate local model training, as shown in (2). In (3), $\left(D_{j}-d_{j}^{e}\left[t\right]\right)$ is the amount of training data, which is required for initiating local model update. Here, when multiple users are associated with an edge, i.e., $x_{\left(i,j\right)}=1$ for a specific $e_{j}$, the number of uploaded training data from the associated users should be larger than the $\left(D_{j}-d_{j}^{e}\left[t\right]\right)$ if $e_{j}$ can initiate the training, i.e., $I_{j}\left[t\right]=1$. On the other hand, if $e_{j}$ cannot initiate the training, the number of uploaded training data from its associated users does not need to exceed $\left(D_{j}-d_{j}^{e}\left[t\right]\right)$. Thus, in the right-hand-side of (3), we need to multiply $I_{j}\left[t\right]$ in order to relax the restriction when $e_{j}$ cannot initiate its own local training, i.e., $I_{j}\left[t\right]=0$. In (4) and (5), the scheduling between $e_{j}$ and $u_{i}$ is expressed, i.e., each $u_{i}$ can be scheduled with one $e_{j}$ (refer to (4)) and each $e_{j}$ can be associated with, at most, $a_{j}$ number of users (refer to (5)).

## 4. Performance Evaluation with Real-World Dataset

This section verifies the novelty of the proposed algorithm with real-world data obtained Keum Rivers in Figure 2. The detailed simulation parameters and the data-intensive simulation results are discussed.

### 4.1. Simulation Setup

The performance of the proposed optimally fair FL scheduling algorithm is evaluated via data-intensive simulations. The simulator is designed with cvxpy [28]. We set the simulation environment as shown in Table 1 by referring to the actual river in Figure 2. Each barrage in the river has 5 edges, which can receive the water quality data from the distributed smart sensors. The smart sensors generate sampling data based on each sampling frequency. We set the smart sensors with different sampling frequencies, which are randomly distributed around the edges. Additionally, the number of antennas for each edge is considered between 30 and 40 based on NB-IoT communications. The sampling data from the smart sensors can be only used as meaningful learning data through edge servers. Each smart sensor sends the data to the scheduled edge only when $x_{\left(i,j\right)}=1$. In the simulation, the edge saves the received data; if the data size is less than *D*, it waits for the next scheduling to meet the size of *D* and if the data size is equal to value of *D* or higher, it uses all received data for local model training.

#### 4.1.1. Water Quality Monitoring Data

We collect the water quality data for the simulation from the State-led managed sites (http://www.koreawqi.go.kr, http://water.nier.go.kr, http://www.wamis.go.kr) (accessed on 1 January 2021). Each site provides information on real-time water quality and green tide outbreak in Korea. The disclosed data from the sites differ from each other in terms of inclusive water quality indicators and measurement time or coordinates. Generally, seven sensing data (i.e., temperature, pH, DO, TOC, TN, Chl-a, and turbidity) are used as water quality monitoring indicators. For these reasons, we collect the seven kinds of cumulative data over 5 years that are provided from different sites and refine the obtained water quality records by pre-processing, considering the missing data, normalization of variables and class imbalance. Through this process, all the essential data are formulated to form of one row in the dataset based on the same date (time) and location, such as in Table 2. The seven indicators’ observation data of one row are defined as one set of training data for the green tide prediction model and as real-time data that are generated repeatedly for the sampling frequency in each smart sensor. The total data for one river can be controlled by considering the deployment value of barrages, edges, and smart sensors or any specific sampling frequency.

#### 4.1.2. ML/DL-Based Prediction Model

The green tide occurrence prediction model used in this paper is a binary classification model based on deep neural networks. Based on the seven water quality indicators data observed in real-time, the model learns the relationship between the indicator values and the possibility of green tide occurrence.

**Figure 5:**
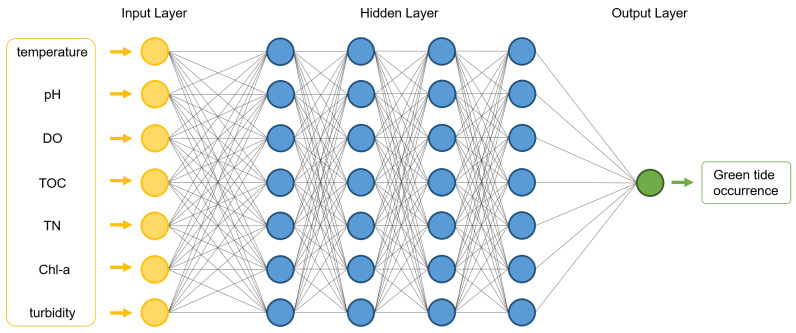
ML/DL-based green tide phenomenon prediction neural architecture.

The network architecture is formulated with 7 inputs parameters (due to seven indicators) and 1 output parameters (due to binary decision). The model includes 4 hidden layers and each layer is followed by ReLU and sigmoid activation functions [29]. For the model training, Adam optimizer is adopted with a learning rate of 0.001, weight decay of 0.0001, and finally the batch size is set to 10. Our proposed binary classification deep neural network model is also illustrated in Figure 5.

In conventional deep neural network computation procedures, we have three phases: (1) model setup (building a deep neural network model), (2) training (optimizing the parameters in our deep neural network model), and (3) inference (making a decision, which is a binary classification decision in this paper, based on the trained/optimized parameters and deep neural network model). For the first phase (i.e., model setup), we already introduced our model, as illustrated in Figure 5, with four hidden layers and each layer has seven units. Furthermore, the sizes of input and output are 7 and 1, respectively. For the next phase (i.e., training), we trained our model, which was built in our first phase with real-world green tide phenomenon data, as explained in Section 4.1.1. Then, the parameters in our proposed deep neural network will be trained and optimized. Then, our learning procedure is over. After that, lastly, our final phase (i.e., inference) is conducted in real-time. When we gather the real-world green tide phenomenon sensing data from smart IoT sensors in real-time, we can make input vectors, and then we can compute output results (between 0 and 1) based on the trained/optimized deep neural network model. If the output result is bigger than 0.5, we can make a decision on whether the green tide phenomenon occurred or not in the position.

### 4.2. Performance Evaluation Results

In this subsection, we describe the simulation results in several respects. The simulation is processed under the simulation settings, already mentioned above.

**Figure 6:**
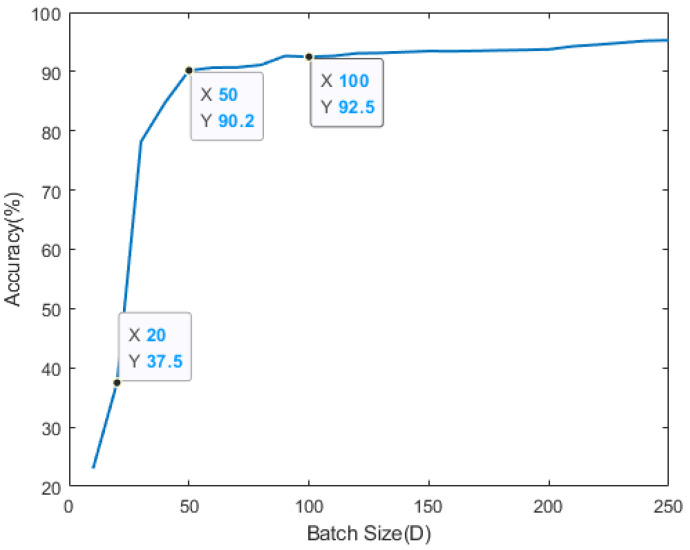
Average local model accuracy with training initiation threshold *D*.

First, we derive the local model performance for each edge of the river, increasing the size of *D* from D=10 to D=250. As the value of *D* becomes larger, the accuracy of the local model, which is trained on one edge is increased. As we can see in Figure 6, the local model’s accuracy rises sharply at the moment where *D* size is between 20 and 50. When *D* size is over 50, the accuracy is slightly higher and is converging to 90% or more from *D* is 100. Because the frequency and number of smart sensors generating water quality observation data in the Keum river are fixed, the total amount of data that exists within the entire system is also defined. Therefore, it is necessary to set the appropriate value of *D* in advance. Based on the result in Figure 6, the simulation is set to target where the local model of each edge guarantees more than 80% accuracy.

We evaluate the scheduling performance based on the number of *I*. The value of *I* indicates whether *I* edges have enough data and are able to learn the local models in the system. As we set the condition in the proposed scheduling algorithm’s equation, only when each edge gets more than *D* amount of data, *I* becomes 1 and the size of *D* guarantees the corresponding local model accuracy. For these reasons, the results represents that the higher the value of *I*, the more edges that have sufficient learning data through the matching methods used in the experiment, and each edge can train the green tide prediction model, which can participate in the global model generation. We proceed with the simulation during 50 iterations and compare the proposed optimally fair scheduling algorithm performance results. Baseline matching (i.e., randomly matched method), which ignores filling *D*, is used as a comparative group.

**Figure 7:**
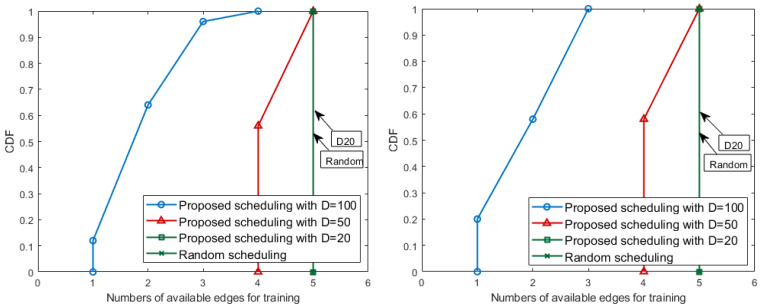
The numbers of available edges for training in Keum River: Baekjae barrage (**left**) and Gongju barrage (**right**).

This evaluation has the performance results, as shown in the Figure 7. The results, based on the size of *D*, are distinguished with different marker types and line colors in Figure 7 and the black dash line with a plus-shape marker represents a baseline. The baseline mechanism, which is a mechanism to let each edge use all the received data, even if the data are too small and unsuitable for local learning, and always has the largest value that is equal to the number of edges in the system. On the other hand, for the proposed matching algorithm, which lets all edges satisfy the *D* size, there are performance differences depending on the value of *D*. In Figure 7, when the training initiation threshold *D* is set 20, the proposed scheduling algorithms always match the baseline performance. The larger the setting for the *D* value, the greater the amount of data that the edge must receive from the sensors to meet the conditions. The size of sampling data produced by the sensors in every iteration is always constant. For these reasons, the number of edges, which satisfy *D* for each period, decreases compared to the results with small *D*. However, even in this situation, the proposed algorithm matches the sensors and the edges in an optimally fair direction and produces as many federated learning participants (i.e., edges) as possible.

**Figure 8:**
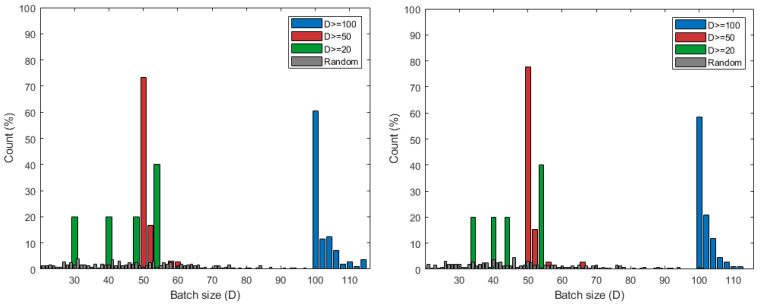
Data distribution of edges over *D* in Keum River: Baekjae barrage (**left**) and Gongju barrage (**right**). The value of Count (%) means the ratio of the number of edges according to the *x*-axis value compared to the cumulative total of edges that satisfy the value D during 50 iterations.

We also measure the number of edges that satisfies the training threshold *D* and the actual data size of the edges over the 50 iteration. Table 3 shows the number of accumulated edges that satisfies the conditions according to the value of *D*, as tested using 5 edges located in Backjae barrage and Gongju barrage. The random matching, which is represented with gray color, results in all edges being able to start local learning with the received data no matter the size. It also has the most variety in the numbers of data distribution. If D=20, 5 edges always meet the threshold during all iterations. If D=50, most edges satisfy the *D* value according to Figure 7, and eventually approximately 220 edges always have more than *D* data. Even if D=100, as seen in Figure 7, only two or three edges meet *D* on average, and less than 50% of the edges of D=20 have 100 or more data. Figure 8 shows the amount of data the edges have based on the cumulative number of edges that meet the *D* condition. For D=20, edges obtained between 30 and 55 pieces of data, and always exceeds the threshold value of 20. For D=50, 75% or more of the cumulative number of edges receive data as much as the value of *D*, and in some cases have more than *D*. If D=100, all of the data on the edge in Figure 8 that are distributed have at least 100 pieces of data applied to approximately 110 cumulative edges as seen in Table 3. About 140 edges, excluding 110 out of 250 total, always receive less than 100 pieces of data and fail to perform the learning.

**Figure 9:**
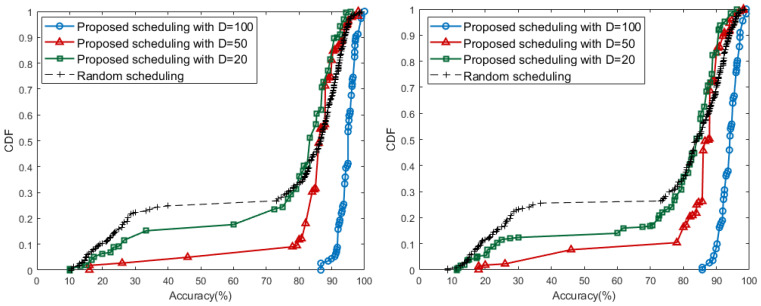
Accuracy CDF of Keum River: Baekjae barrage (**left**) and Gongju barrage (**right**).

Model accuracy is also tested using real data received by the scheduling results above *D*. We derive CDF graph for the performance of the local model generated by each edge. In Figure 9, the results of different *D* sizes are distinguished by the color of the line and the shape of the marker. One marker represents the cumulative distribution of model accuracy corresponding to the *x*-axis. Both Backje barrage and Gongju barrage have a concentration between 90 and 100 accuracy at D=100, and have a relatively even distribution compared to other cases. For D=20, there are models with an accuracy of less than 40, which matches the data distribution between 30 and 50 of Figure 8. At D=50, the models generally have performance accuracy of above 80 and, in particular, the number of edges where the accuracy increases rapidly in 8090 performance range.

Consequently, the results of the proposed scheduling algorithm show that the case of D=50 satisfies the appropriate balance between the number of local learning available edges and accuracy. In Table 4, when *D* has value of 50, the largest number of edges during all iterations satisfy target performance. When *D* has a small value such as 20, the proposed scheduling with D=20 has a similar ratio to the random scheduling in Table 4. Even if almost all edges are allowed to start training, the performance of edges being more than 80% is not always guaranteed. The experiments also present that, if *D* is too large, high performance models can always be obtained. However, in this case, each edge should receive more than the threshold value *D* and it is impossible to get many edges that can perform learning.

## 5. Conclusions and Future Work

Nowadays, various deep learning algorithms are widely used for major environmental problems, and this paper designs a novel green tide phenomenon prediction model which is one of major environmental problems. In order to gather the green tide related indicator information in real-time, smart sensors are used and the gathered data will be delivered to intermediate edge servers (not directly to a centralized computing platform) due to the limitations of transmission distances and the burdens of massive accesses. Then the edge servers conduct their own local training, and the trained parameters are delivered to the centralized platform for building our desired global prediction model via federated learning computation. In order to improve the performance of federated learning, gathered data should be fairly distributed over the edges’ servers for avoiding overfitting. Therefore, this paper proposed a novel federate learning scheduling algorithm in order to fairly schedule sensing data over edge servers. Our data-intensive simulation results verify that the proposed algorithm presents remarkably effective performance due to balanced data gathering. As future research directions, various factors, such as the positions of network components (i.e., smart sensors, and edges), distribution ratios, and communication channel qualities can be considered. In addition, experiments can be extended using various real-world data for a wider range of regions.

## Figures and Tables

**Table 1:** Basic network component statistics in Keum river.

	Keum
# barrages	2
# edge servers for 1 barrage	5
# sensors for 1 barrage	144

**Table 2:** Water quality monitoring data sample of Keum River with 7 indicators. The sample data are based on the data observed around Backjae barrage and Gongju barrage.

Location (Barrage)	Temperature	pH	DO	TOC	TN	Chl-a	Turbidity	Green Tide Occurrence
Backjae	17.7	7.6	8.9	3.5	3.042	6.1	8.8	0
Backjae	23.1	7.4	8.6	3.5	2.089	17.6	7.4	0
Backjae	27	8.4	10.5	3.3	2.753	45.8	12.7	1
Backjae	27	8.6	10.8	3.5	2.586	67.1	20.3	1
Backjae	27.3	8.4	9.3	3.2	2.889	192.8	5.9	1
Gongju	26.4	7.7	8.8	3.3	2.636	6.7	2	0
Gongju	24.5	7.6	8.9	2.8	2.994	9	4	0
Gongju	27.4	8.1	9.4	2.3	2.428	44.1	10.6	1
Gongju	27.9	8.1	8.8	2.7	2.77	48.7	6.3	1
Gongju	30.9	8.7	9.3	3.2	1.995	67.2	5.6	1

**Table 3:** Cumulative total values of edges that satisfy the training threshold *D*.

	Baekjae Barrage	Gongju Barrage
*D* = 20	250	250
*D* = 50	222	221
*D* = 100	114	111

**Table 4:** Achieved accuracy ratio of Kuem River (>=80 total percentage).

	***D* = 20**	***D* = 50**	***D* = 100**	**Random**
Baekjae barrage	172	201	114	161
	250	222	114	250
	0.688	0.905	1.000	0.644
	***D* = 20**	***D* = 50**	***D* = 100**	**Random**
Gongju barrage	166	198	111	165
	250	221	111	250
	0.664	0.896	1.000	0.660

## Data Availability

Data sharing not applicable.
